# Field Cage Studies and Progressive Evaluation of Genetically-Engineered Mosquitoes

**DOI:** 10.1371/journal.pntd.0002001

**Published:** 2013-01-17

**Authors:** Luca Facchinelli, Laura Valerio, Janine M. Ramsey, Fred Gould, Rachael K. Walsh, Guillermo Bond, Michael A. Robert, Alun L. Lloyd, Anthony A. James, Luke Alphey, Thomas W. Scott

**Affiliations:** 1 Department of Entomology, University of California Davis, Davis, California, United States of America; 2 Istituto Pasteur-Fondazione Cenci Bolognetti, Università la Sapienza, Rome, Italy; 3 Centro Regional de Investigación en Salud Pública, Instituto Nacional de Salud Pública, Tapachula, Chiapas, México; 4 Department of Entomology, North Carolina State University, Raleigh, North Carolina, United States of America; 5 Fogarty International Center, National Institutes of Health, Bethesda, Maryland, United States of America; 6 Biomathematics Graduate Program and Department of Mathematics, North Carolina State University, Raleigh, North Carolina, United States of America; 7 Departments of Microbiology and Molecular Genetics and Molecular Biology and Biochemistry, University of California Irvine, Irvine, California, United States of America; 8 Oxitec Ltd., Abingdon, Oxfordshire, United Kingdom; 9 Department of Zoology, University of Oxford, Oxford, United Kingdom; Centers for Disease Control and Prevention, United States of America

## Abstract

**Background:**

A genetically-engineered strain of the dengue mosquito vector *Aedes aegypti*, designated OX3604C, was evaluated in large outdoor cage trials for its potential to improve dengue prevention efforts by inducing population suppression. OX3604C is engineered with a repressible genetic construct that causes a female-specific flightless phenotype. Wild-type females that mate with homozygous OX3604C males will not produce reproductive female offspring. Weekly introductions of OX3604C males eliminated all three targeted *Ae. aegypti* populations after 10–20 weeks in a previous laboratory cage experiment. As part of the phased, progressive evaluation of this technology, we carried out an assessment in large outdoor field enclosures in dengue endemic southern Mexico.

**Methodology/Principal Findings:**

OX3604C males were introduced weekly into field cages containing stable target populations, initially at 10∶1 ratios. Statistically significant target population decreases were detected in 4 of 5 treatment cages after 17 weeks, but none of the treatment populations were eliminated. Mating competitiveness experiments, carried out to explore the discrepancy between lab and field cage results revealed a maximum mating disadvantage of up 59.1% for OX3604C males, which accounted for a significant part of the 97% fitness cost predicted by a mathematical model to be necessary to produce the field cage results.

**Conclusions/Significance:**

Our results indicate that OX3604C may not be effective in large-scale releases. A strain with the same transgene that is not encumbered by a large mating disadvantage, however, could have improved prospects for dengue prevention. Insights from large outdoor cage experiments may provide an important part of the progressive, stepwise evaluation of genetically-engineered mosquitoes.

## Author Summary

The absence of a commercially-available dengue vaccine or anti-viral drug makes control of *Aedes aegypti*, the principal dengue mosquito vector, the only available method to prevent this disease. Sustained, effective application of vector control, however, has been difficult and this led to the call for innovative strategies, including genetic approaches. Here, the authors investigated the ability of a genetically-engineered strain of *Ae. aegypti* to eliminate wild mosquito populations in large outdoor cages. Females of the engineered strain cannot fly and, therefore, cannot mate or take blood meals necessary to lay viable eggs. Wild females that mate with genetically-engineered males, therefore, will not produce reproductive female offspring. In this study, although population reductions were detected in 4 of 5 field cages, none of the wild mosquito populations were eliminated. A mating disadvantage for genetically-engineered males appeared to account for a significant part of their fitness disadvantage. Results suggest that this specific strain may not be effective in a large-scale release and that new strains with the same or similar transgene, but improved mating performance, may be more effective for preventing dengue. Results also indicate that large outdoor cage experiments may provide valuable insights into the progressive, stepwise assessment of genetically-engineered mosquitoes.

## Introduction

The recent worldwide increase in dengue [1], [2] has made urgent the development and assessment of new tools for controlling the disease [3]. Because no vaccines or drugs are commercially available [4], [5], mosquito vector control by insecticides, insect growth regulators and larval development site elimination (source reduction) are the current means for dengue prevention [6]. Long-term control of *Aedes aegypti*, the most efficient dengue vector [7], is a challenging and expensive task that is difficult to achieve and maintain, especially in developing, resource-challenged environments [8]–[10]. Genetically-engineered (GE) *Ae. aegypti* strains that are unable to transmit dengue [11] or that bear sterility genes [12], [13] constitute new tools to control dengue and merit confined experimental evaluation while public and scientific discourse enables appropriate oversight of this new technology [14], [15]. Concern regarding the use of GE organisms, and the absence of guidelines to help researchers interact with local communities, motivated the elaboration of a framework for the development, evaluation, and application of genetic strategies for prevention of mosquito-borne disease [16]. These guidelines were followed carefully in the development and execution of the experiments described here.

The OX3604C strain of *Ae. aegypti* contains a tetracycline-regulated transgene that induces a female-specific flightless phenotype that cannot reproduce as a consequence of its inability to fly and mate [17]. Tetracycline is added to larval rearing water to allow normal female development during colony maintenance and amplification, but is not added during the generation used for mass-production of males. This enables genetic removal of females, because females carrying the transgene and reared in the absence of tetracycline cannot fly. Similarly, female offspring that result from matings between wild-type females and released OX3604C males are unable to fly or reproduce. The goal in releasing OX3604C males is to control dengue virus transmission by reducing or eliminating *Ae. aegypti* populations. The release of insects carrying a dominant female-lethal construct has four main advantages compared to a traditional sterile insect technique (SIT): (i) no need to sort males and females, (ii) no need for facilities to irradiate males, (iii) the transgene has an effect in subsequent generations because it is dominant and inherited by male offspring, and (iv) OX3604C contains a heritable, fluorescent marker (DsRed2) that distinguishes it from immature wild-type *Ae. aegypti* [17].

As an initial step in OX3604C evaluation, transgenic males were introduced weekly at an 8.5–10∶1 OX3604C∶target ratio into large laboratory cages with constant temperature, humidity, and photoperiod, that contained stable target populations of wild type *Ae. aegypti* [18]. Target populations were eliminated in all experimental cages in 10–20 weeks, supporting further analyses of this strain in contained or confined field trials to evaluate mating competitiveness and environmental and other effects on its performance [18].

Progressive evaluation of OX3604C from laboratory to field cages prior to open field release is valuable because it allows for systematic assessment of possible environmental effects on mosquito performance under conditions increasingly more natural. Comparison of transgenic mosquito performance in laboratory versus semi-field conditions is expected to provide valuable data for planning subsequent experimental assessments and refine strategies for disease prevention. Insectary studies in a laboratory, field cage experiments and deliberative community engagement activities are all part of the progressive transition of engineered insects from the laboratory to open field releases [19], [20]. This is particularly true when the transgene as well as all other genes in the transgenic strain can be introduced into natural target populations and transmitted to subsequent generations. Even though OX3604C is a self-limiting strategy that lacks a gene drive component, it can introduce through heterozygous males new alleles and genes into target populations.

We report the effect of OX3604C in reducing target *Ae. aegypti* populations in the first large outdoor field cage trial of a transgenic population suppression tool. While density reduction was significant in four of five target populations, we did not observe population elimination in any of the cages within the time expected. A series of subsequent experiments revealed a significant mating disadvantage for OX3604C males that was not observed in the laboratory study. We discuss the implications of our results for OX3604C and more broadly for future evaluations of genetically-engineered mosquitoes.

## Materials and Methods

### Field site

Our study was carried out on a plot of land (14°51′41″N, −92°21′15″W) referred to hereafter as the “field site.” The land was a 4.5 ha flat, rural area located 11.2 km southeast of the center of Tapachula, Mexico, in the village of El Zapote (Ejido Rio Florido). The study area is characterized by a tropical climate with a rainy season from May to October (average total rainfall of 2,100 mm) and a dry season from November to April (average total rainfall of 50 mm). Supportive laboratory and insectary facilities were located at Centro Regional de Investigación en Salud Pública (CRISP), Tapachula, 15 km from the field site.

The protocol used in this study was similar to that used during the previous OX3604C assessment in laboratory insectary conditions [18]. Materials and methods were adapted to different logistics and biosecurity conditions required for a field-cage experiment. The most important differences between laboratory and field-cage experiments are summarized in Table S1. Mexico has a mature regulatory system for the use of genetically modified organisms, having a law and implementing regulations in place since 2005. Field cage experiments must comply with basic biosafety procedures, oversight, and registration of all experimental procedures, installations and monitoring programs. Our protocol was approved by the Mexican institutions Instituto Nacional de Salud Pública (#581) and Secretería de Medioambiente y Recursos Naturales (S.G.P.A./DGIRA/DG/7074/09).

### Ethics statement

This study was carried out in strict accordance with the recommendations in the Guide for the Care and Use of Laboratory Animals of the National Institutes of Health. Protocols were approved by the Institutional Animal Care and Use Committee of the University of California, protocol 15653 [UCD] and the Instituto Nacional de Salud Pública INSP Biosecurity permit #581 [CRISP].

### Cage design

Our semi-field system consisted of six caging units each measuring 6×6×2 m (LxWxH) (Figure S1). A solid plastic roof with sunscreen around the edges covered all six cages, and provided shade and protection from direct sunlight. One caging unit was used as a field laboratory for larval rearing and adult mosquito handling. Each of the other five caging units was divided in half by means of zippered mesh walls resulting in a total of 10 6×3×2 m cages (five pairs) (Figure S2). Each pair was provided with two vestibules that allowed access to both cages through three sleeves, two of which opened to shelves inside the cage and a third opening to the cage floor (Figure S3). Cages were made of white tricot mesh reinforced with white fabric and located on a platform elevated 1 m above the ground, with ∼3–5 m between the top of each cage and the roof covering the platform. Materials and general design used to build the cages is described in detail by Facchinelli *et al.* [21]. Temperature and relative humidity were measured by means of data-loggers (Hobo Pro v2 temp/RH, Onset Computer Corporation, Bourne, MA), located inside each cage and in the outdoor environment, 30 meters from the cages.

### Biosecurity procedures

The protocol for field cage design, OX3604C colony maintenance, and field cage experiments was approved by the Instituto Nacional de Salud Pública (INSP) and Secretaría de Medio Ambiente y Recursos Naturales (SEMARNAT), Mexico, under the provisions of the law on genetically modified organisms (Ley de Bioseguridad de Organismos Genéticamente Modificados, (Marzo 2005)). The field cage experiment protocol included procedures for detection of potential escapes of OX3604C mosquitoes to the open environment. Ten ovitraps and 10 BG-Sentinel Mosquito Traps (Biogents AG, Regensburg, Germany) were distributed on the ground around the cage platform. Three BG Sentinel Traps were located inside the CRISP insectary where larvae were reared. Ovitraps were serviced weekly and collected eggs were hatched and larvae screened for the fluorescent marker. BG Sentinel Traps were checked daily and *Ae. aegypti* adults were processed by gene amplification (PCR) for transgene detection. No transgenic mosquitoes were collected outside the field cages at the field site. Two transgenic adults were collected by BG Sentinel Traps in the room dedicated to OX3604C colony maintenance in the CRISP insectary.

### Mosquito strains

The OX3604C strain employed in this study was obtained by backcrossing the homozygous OX3604C strain [17] into the genetic background of the genetically-diverse laboratory strain #1 (GDLS1; [18]) derived from 10 geographically-distinct populations collected during 2006 in Chiapas, Mexico [22]. Approximately 96.9% of the genome not linked to the transgene is expected to consist of GDLS-derived sequences [18]. Before introducing OX3604C into treatment cages, it was determined that the strain was not homozygous for the transgene. Screening for the fluorescent marker showed that the wild-type allele had a frequency ranging between 4.7 and 8.7%. A mathematical model indicated that at the release ratios we used, this low level of wild-type alleles was not expected to significantly affect the outcome of the experiment (Figure S4 and S5). Wild-type females were removed prior to introducing OX3604C males into treatment cages.

The target and control *Ae. aegypti* populations in our study were the genetically-diverse laboratory strain #2 (GDLS2) derived from 2008 field collections in the same locations in Chiapas where GDLS1 originated [18]. *Aedes aegypti* females were allowed to imbibe blood directly from rabbits for colony maintenance, field cage equilibration, and experiments (UC Davis Animal Care and Use protocol 15653 and INSP Biosecurity permit #581).

### Stabilization of target population

Target and control populations were established in each of the 10 experimental cages during a 16-week period using GDLS2 mosquitoes. Shelves inside each cage held (i) a total of four plastic oviposition containers filled with ∼600 ml sterilized water and were lined with filter paper, (ii) four plastic larval development containers filled with ∼600 ml sterilized water and enclosed in a mesh-covered dome to prevent oviposition, and (iii) four plastic plates containing raisins as a source of sugar for adults (Figure S3). Each cage also contained two humid, adult resting sites; i.e., 15 L black plastic buckets that were lined with black fabric and contained a mesh-covered water container.

GDLS2 eggs were hatched in Centro Regional de Investigación en Salud Pública (CRISP) insectary and larvae transported to the field site after 48 hours. Establishment of the target population was initiated by adding 300 second-instar GDLS2 larvae weekly in each cage from week 0 to week 3. Larvae were fed dried brewer's yeast *ad libitum* and females were fed blood from restrained rabbits once a week for 30 minutes. From week 4 to week 16, the target and control GDLS2 populations were maintained by returning eggs laid by females in each cage to their respective cages as second-instar larvae at a rate of 200/week. Eggs produced in cages were collected twice a week and adults were sampled weekly to monitor population dynamics. Eggs were transported to the insectary, counted, dried and stored in a humidified chamber, and then hatched for the next generation. Adults were sampled using BG Sentinel Traps placed in each cage for 30 minutes each week, counted, sexed after sedation on a CO_2_ sedation device (Figure S6), and returned to their respective cage. Adult trapping and handling resulted in 0–8% mortality. Cages were inspected at least weekly for the presence of spiders, ants or other mosquito predators. When arthropod predators were found they were removed mechanically, without the use of insecticides. No vertebrate predators were found during the 33-week period that mosquitoes were in field cages.

### Introduction of OX3604C

The lack of a homozygous OX3604C strain prevented us from rearing and allowing OX3604C mosquitoes to emerge into treatment cages as was performed by Wise de Valdez *et al*. [18]. Instead, pupae were sexed by visual examination for size at the field laboratory insectary using 3 ml plastic droppers and only adult males were added into treatment cages to avoid introducing the few females lacking the transgene (∼0.5%) that could have interfered with population extinction. OX3604C eggs were hatched weekly without tetracycline in the CRISP insectary, second-instar larvae were transported to the field laboratory insectary, placed in 35 plastic trays (each tray contained 500 larvae in 1.5 L of water) and fed dry ground yeast *ad libitum*. Pupae were collected over the course of three days and sexed so that a total of 5,250 male pupae were introduced into the five-insectary cages (1,050 per 30×30×30 cm cage, taking into account 5% mortality), which were held in the field laboratory insectary. The sex of recently emerged adults was checked daily by visual examination of adults in cages, females were removed, and males were introduced into their respective treatment cages every 24 h over the course of 4 days each week. The release number remained constant. During the first OX3604C male release [week 0 post-release], the release ratio was approximately 10 times the weekly return rate of 200 second-instar/GDLS2 larvae/week (i.e., approximately 100 GDLS2 males). Because the mosquito population tended to decreased over time in the treatment cages, the release ratio correspondingly increased from 10⋮1 OX3604C⋮target males during Week 0 post-release to between 14⋮1 to 1,000⋮1 during Week 17 post-release (Figure S7). During Week 1 and 2 post-release, a total of 63 GDLS2 males from control cages and 50 OX3604C males that emerged from rearing trays at the field site were collected to compare size of the two strains. Right wing measurements were used to determine mosquito body size.

### Population maintenance post OX3604C introduction

Starting on week 0 post-release, a 10% sample of eggs produced in each treatment cage was screened weekly for the DsRed2 marker to assess transgene introgression into target populations. When the first fluorescent larvae were detected in treatment cages confirming that OX3604C males were mating with GDLS2 females (week 3 post-release), the number of larvae added back to each cage was adjusted to reflect the impact of the OX3604C male release on egg production. Briefly, the number of larvae returned to control cages remained constant at 200 second-instar larvae/week, while the number of larvae added back to each treatment cage was changed to a proportion of the egg number in the respective paired control cage based on the procedures applied in Wise de Valdez *et al.* [18]. At the end of our experiment, week 18 post-release, all mosquitoes present in the field cages were collected with a backpack aspirator (John W. Hock Company, 23^rd^ Ave, Gainesville, 32606 Florida, U.S.A.).

### Mating competitiveness

A total of six mating competition experiments were carried out between December 2010 and June 2011 with the aim of investigating the lower performance of OX3604C in the field cages trial versus the laboratory insectary trial [18].

#### Experiments 1, 2 and 3

Four insectary cages located at the CRISP insectary (two 60×60×60 cm and two 30×30×30 cm), and four cages located in the field laboratory (two 60×60×60 cm and two 30×30×30 cm), were each populated with 10 GDLS2 females, 10 GDLS2 males and 10 OX3604C males, all 3–4 days post adult eclosion. To populate the cages, virgin males were introduced first, followed by females so that females had equal opportunity to mate with GDLS2 or OX3604C males. This procedure also was used during mating competition Experiments 4–6. Males and females were housed together for 24 hours after which females were offered blood from rabbits for two consecutive days. Each female was transferred into a mesh-screened plastic cup lined with humid filter paper to stimulate oviposition. Eggs collected from each female were conditioned and then hatched at the CRISP insectary. Second- and third-instar larvae were screened for DsRed2 marker fluorescence.

#### Experiment 4 (two replicates)

Three 30×30×30 cm cages housed in the field laboratory were each populated with 10 GDLS2 females, 10 GDLS2 males, and 10 OX3604C males. At the same time, five large field cages that had been used in the population reduction experiment were each populated with 100 GDLS2 females, 100 GDLS2 males, and 100 OX3604C males. Males and females were housed together for 24 hours after which females were offered blood from rabbits for two consecutive days. All females from the small cages and 20 females from each large field cage were transferred individually into a mesh-screened plastic cup lined with humid filter paper to stimulate oviposition. Eggs collected from each female were counted, conditioned and then hatched at the CRISP insectary. Second- and third-instar larvae were screened for fluorescence.

#### Experiment 5

Eight 30×30×30 cm cages, four located in the field laboratory and, in a deviation from experiment 4, four located in the CRISP insectary, were each populated with 10 GDLS2 females, 10 GDLS2 males, and 10 OX3604C males. At the same time four large field cages were populated with 100 GDLS2 females, 100 GDLS2 males and 100 OX3604C males each. Males and females were housed together for 24 hours after which females were offered blood from rabbits for two consecutive days. All females from the small cages and 20 females from the large field cages were transferred individually into a mesh-screened plastic cup lined with humid filter paper to stimulate oviposition. Eggs collected from each female were counted, conditioned and then hatched at CRISP insectary. Second- and third-instar larvae were screened for fluorescence.

#### Experiment 6 (two replicates)

Six large field cages were used in two replicate experiments for three different treatments: (i) two randomly-selected field cages were populated with 100 GDLS2 females and 100 GDLS2 males that were 3–4 days old (young males and females) and 1,000 OX3604C males that were 10 days old (old males); (ii) two randomly-selected field cages were populated with 100 GDLS2 females, 100 GDLS2 males, and 1,000 OX3604C males that were all 3–4 days old (young mosquitoes); and (iii) two randomly-selected field cages were populated with 100 GDLS2 females, 100 GDLS2 males, and 1,000 OX3604C males that were 9–10 days old (old mosquitoes). Different aged males were used to explore possible effects of male age during field cage experiments when GDLS males that were released into field cage daily and OX3604C males that were released only 3 days per week. Males and females were housed together for 24 hours after which females were offered blood from rabbits for two consecutive days. Thirty females were transferred individually into a mesh-screened plastic cup lined with humid filter paper to stimulate oviposition. Eggs collected from each female were counted, conditioned and then hatched at CRISP insectary. Second- and third-instar larvae were screened for fluorescence

### Statistical analysis

#### Main cage experiment

Distribution of eggs produced weekly in cages was checked for normality using the Kolmogorov-Smirnov test. Data were normalized by square-root transformation prior to performing one-way analysis of covariance (ANCOVA) to detect an OX3604C effect in treatment cages by comparing weekly egg production in paired cages.

The Mann-Whitney U test was used to compare the number of males collected in treatment cages with the number of males collected in the respective paired control cages and the size of OX3604C *vs*. GDLS2 males during the first 2 weeks of OX3604C introduction in treatment cages.

Estimated fitness costs for OX3604C males in each treatment cage were calculated by fitting the model [23] to field data (i.e., frequency of DsRed2 marker in larvae produced in treatment cages, number of target larvae reintroduced weekly in each treatment cage), using a nonlinear least squares procedure. To do this, the mean of 1,000 simulations was fitted by minimizing the sum of the squares of the residuals for the 15 weeks of DsRed2 frequency data for each cage. Simulations were run with the following parameter values: baseline larval input for control cages = 200; release ratio OX3604C∶GDLS2 males = 10; daily female survival probability = 0.90; daily male survival probability = 0.72; mean daily number of offspring per female = 10. The model assumes complete penetrance of the dominant flightless trait. The estimated extinction time for each treatment cage was calculated using the estimated fitness costs and 1,000 simulations of the model [23] for each cage. The extinction time estimates were calculated assuming the input of a 93.3% homozygous strain to account for the average lack of homozgyosity observed in cage experiments.

#### Mating competitiveness experiments

A replicated G-Test (Sokal and Rohlf 1995) was used to investigate if there was an overall significant mating disadvantage to the OX3604C strain in mating competitiveness experiments. To calculate OX3604C mating disadvantage from mating competition experiments, the number of GDLS2 batches was multiplied by OX3604C∶GDLS2 ratio to obtain expected number of OX3604C egg batches. Then the actual number of OX3604C batches was divided by the expected number to obtain a fractional value for the fitness of the OX3604C males. The fractional value for the fitness of the OX3604C males was subtracted from 1 to estimate the mating disadvantage.

## Results

**Figure 1:**
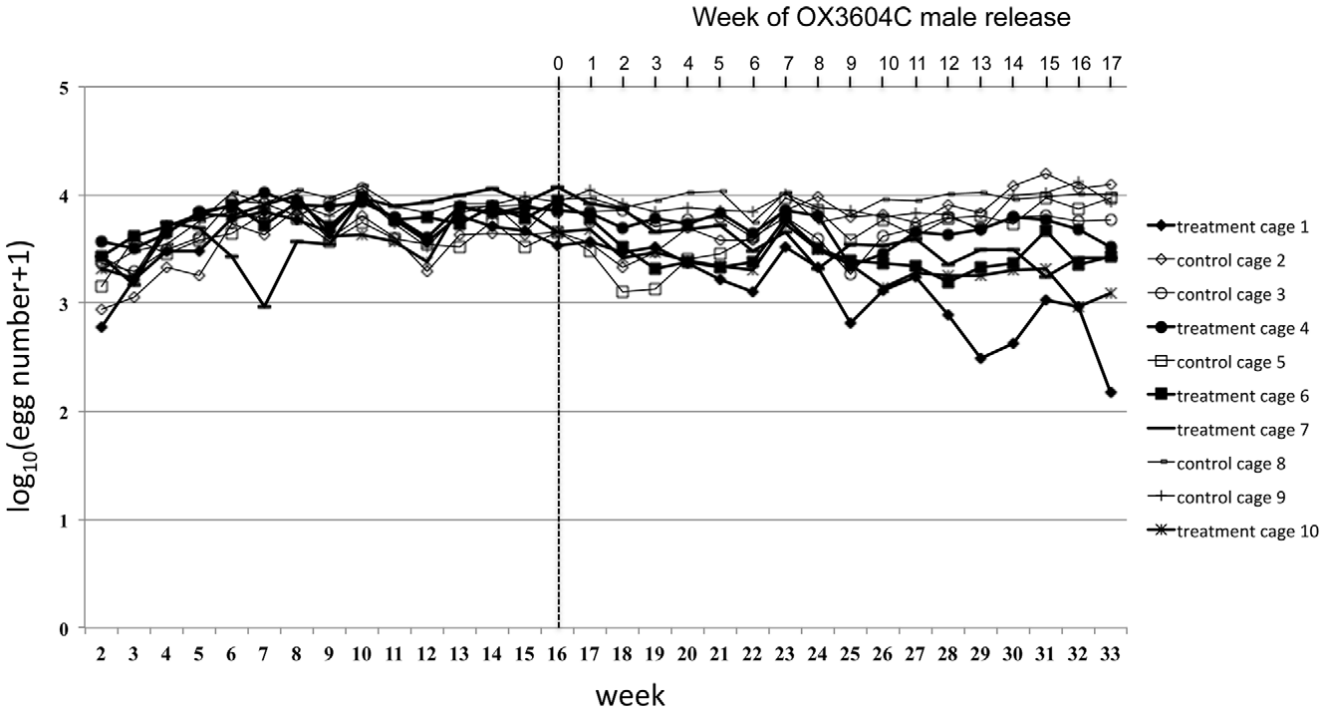
Egg production in treatment and control cages. Weekly egg production is shown for each control and treatment cage. Production numbers were stable in all cages by week 9 after population establishment. After OX3604C male release was initiated (vertical dashed line) in the treatment cages (week 16; week 0 PR, top time axis), egg production in the control cages continued to be stable and declined slightly in the treatment cages.

**Figure 2:**
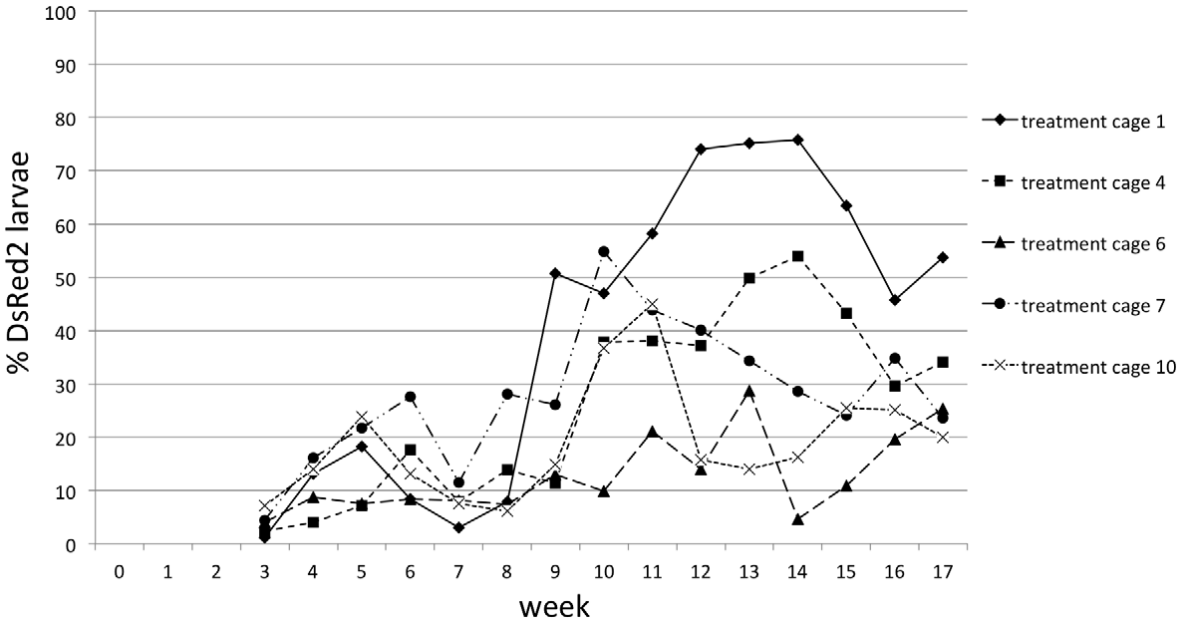
Progeny genotypes in treatment cages. A random sample of eggs from each treatment cage collected weekly was hatched and screened for the DsRed2 marker starting from Week 0 post-release. The number of screened larvae corresponded to 10% of the eggs produced weekly per cage or a minimum of two hundred, when available.

Each of five caging units was partitioned into two paired cages (pair A consists of cages 1 and 2, pair B consists of cage 3 and 4, *etc*.) with dimensions of 6×3×2 meters (LxWxH) (Figure S1 and S2). Populations of the GDLS2 [18] were established in the ten cages for a period of 16 weeks (from April to August 2010) prior to the release of OX3604C males. Population densities stabilized in all cages by week 9 (Figure 1). One cage in each pair was assigned randomly during week 16 as a control or treatment cage (Figure S2). From week 16 to week 33 (from 16 August to 23 December 2010), corresponding to week 0 through week 17 post-release, ∼1,000 OX3604C males were introduced weekly into each treatment cage. This number corresponds to an approximate initial 10∶1 OX3604C∶GDLS2 male release ratio (Figure S7). The constant release number of OX3604C males, calculated to establish the initial 10∶1 ratio based on input rate by the average lifespan, was maintained from week 0 to week 17 post-release. When transgene introgression into the caged populations was first detected (presence of the DsRed2 marker gene in larvae), during week 3 post-release (Figure 2), the weekly number of larvae returned to each treatment cage was adjusted relative to the weekly return rate in the respective paired control cage (held constant at 200 second-instar larvae/week in all control cages). This was done to reflect any impact of OX3604C male release on egg production by females in each treatment cage. Based on the number of larvae returned to treatment cages from week 3 to week 17 post-release, release ratios of OX3604C∶target males increased in all treatment cages reaching the highest value of 1,000∶1 in cage 1 during week 17 post-release (Figure S7).

Temperatures during the trial ranged between 14.5°C and 41.8°C in all cages except cage 6 where a peak of 44.2°C was recorded on 27 April (Table S2). During the rest of the trial, temperatures in cage 6 were similar to those recorded in the other field cages. Daily temperature fluctuations ranged between 2.0°C and 20.7°C. Relative humidity (RH) in field cages ranged from 38.1% during the warmer hours of the day to 99.4% at dawn. Temperatures recorded outside of the cages were similar to those recorded in cages and ranged from 15.8°C to 40.7°C. RH outside of cages also was similar to that recorded inside cages, ranging from 42.8% to 100% with a mean of 89.2%±11.6% (SD) (Table S2).

**Figure 3:**
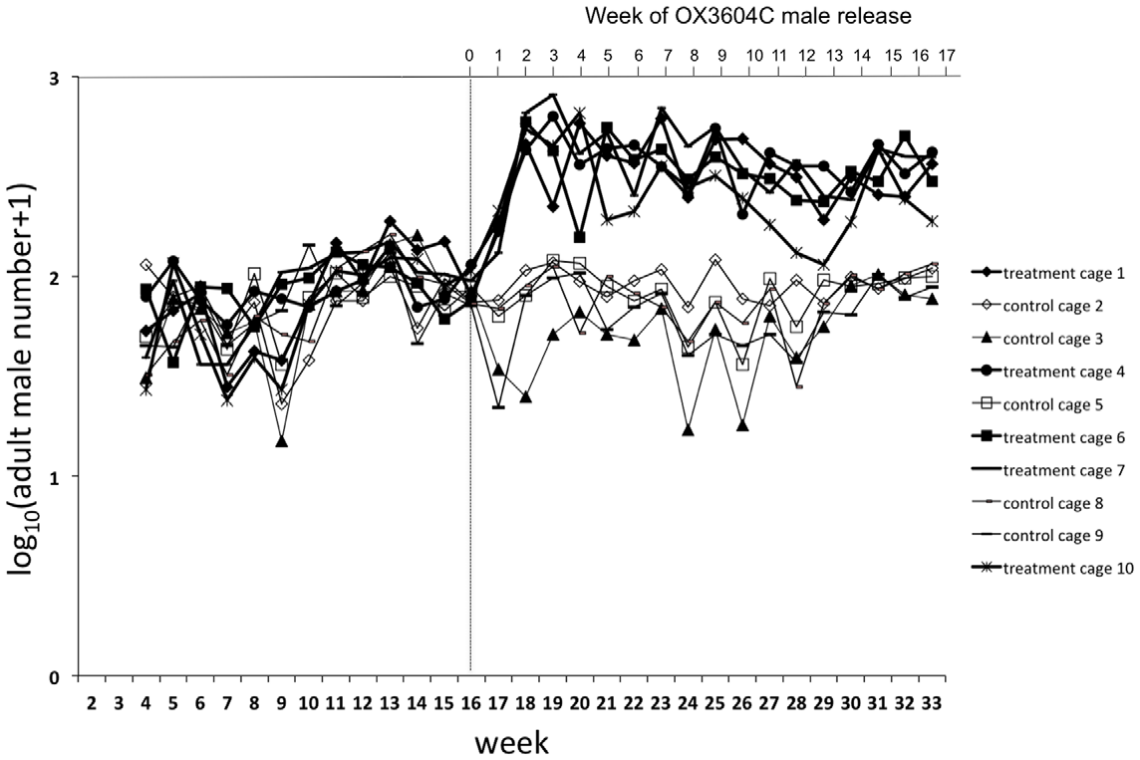
Adult males sampled weekly with BG Sentinel Traps. Each week, adult sampling was performed the day before the weekly release of OX3604C males. Significantly higher numbers of males were collected in treatment cages with respect to control cages (Table S3) starting from Week 2 post-release (PR), indicating that transgenic males were present in large numbers over time in release cages.

Weekly adult sampling performed the day before starting the next OX3604C release into treatment cages confirmed the presence of a significantly higher number of males in treatment cages compared to their respective control cages (Mann-Whitney U test *p*<0.01 for all cage units) (Figure 3). The percentage of DsRed2 larvae produced in treatment cages fluctuated between 1 and 76% but never reached 100% (Figure 2).

**Figure 4:**
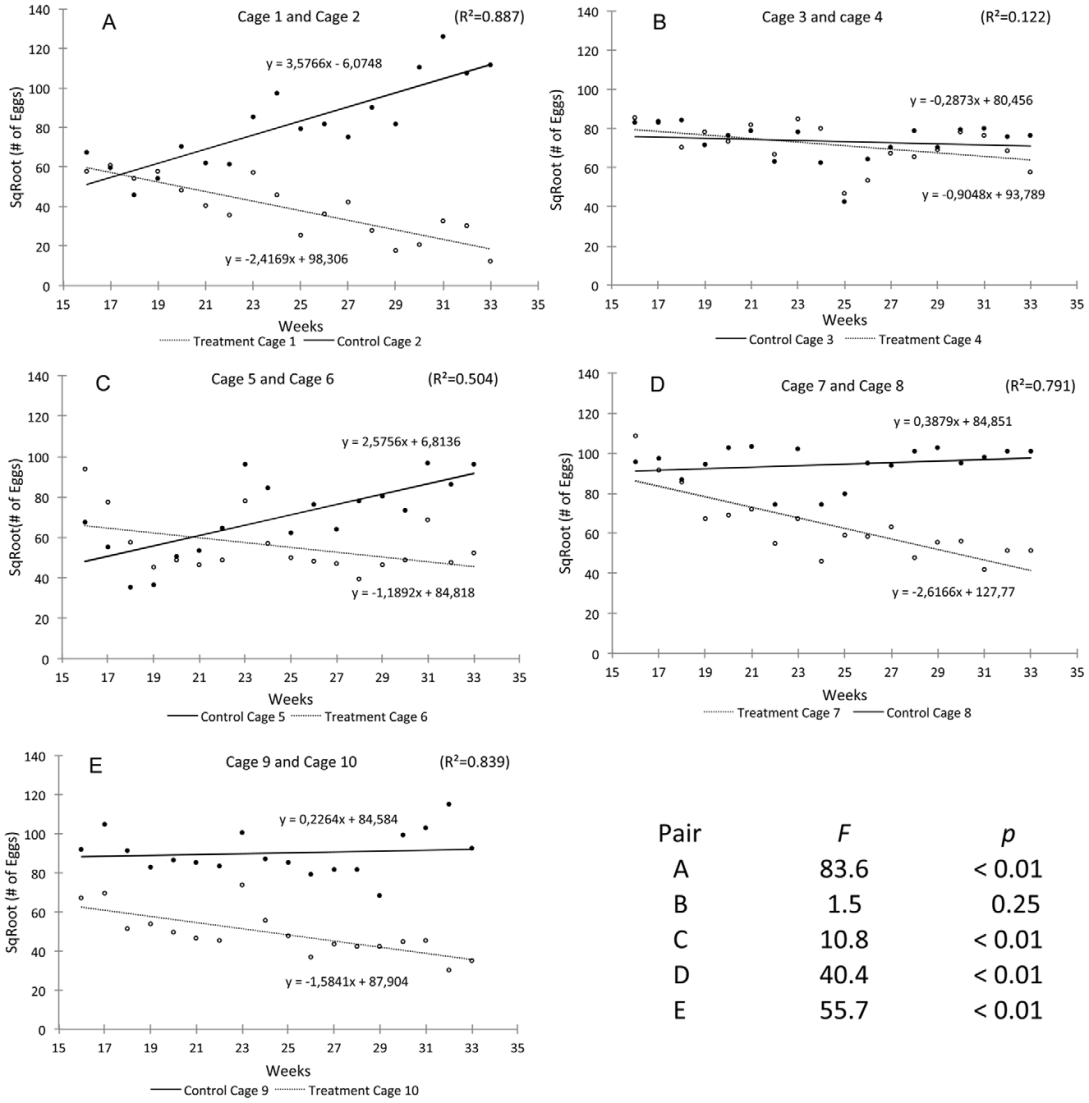
ANCOVA results for egg production dynamics in paired cages A, B, C, D, and E.

ANCOVA indicated that the number of eggs produced in treatment cages decreased significantly subsequent to male OX3604C release compared to respective paired control cages in all treatment cages except cage 4, where covariates did not indicate an effect (Figure 4). Ratios of females collected in control *vs.* treatment cages per each cage unit matched results of ANCOVA (*F*) (Table S3; Figure 4), being highest in pair A and lowest in pair B, following the same ranking (i.e., pairs A, E, D, C, B from highest to lowest values).

No extinction, defined as two weeks without eggs collected in oviposition containers and no adult females collected with BG-Sentinel Mosquito Traps was detected in any treatment cage during the 17 weeks post-release. The low effectiveness of the OX3604C strain in all treatment cages is supported by high OX3604C fitness cost estimates calculated with a simulation model for each treatment cage (Table 1). Model predictions based on extrapolation from the 17 weeks of data indicate that mosquitoes in all but one cage had at most an 11% chance of extinction within 10 weeks from the day the field cage experiment was terminated (probability of extinction for cage 1 was 0.36). On average, predicted extinction times for cages ranged between week 23 and week 65 post-release (Table 2).

**Table 1:** OX3604C fitness cost estimates during the field cage trial per treatment cage.

Cage	Fitness Cost	Sum of Square Error^1^
1	0.9489	0.5023
4	0.9705	0.1848
6	0.9876	0.0517
7	0.9720	0.1717
10	0.9825	0.1479
Mean	0.9723	
SD^2^	0.0149	

1 Sum of square errors associated with each estimate.

2 Standard deviation.

**Table 2:** Extinction time estimated per each treatment cage assuming the estimated fitness costs from Table 1.

Cage	Minimum^1^	Maximum^2^	Mean^3^	SD^4^	Probability of Extinction in Weeks 18–28 PR^5^
1	20	53	29.9	4.515	0.36
4	21	54	35.0	5.722	0.11
6	27	78	43.6	7.830	0.01
7	24	65	36.1	5.664	0.05
10	24	74	40.4	7.105	0.03
Mean	23.2	64.8	37.0		
SD^4^	2.775	11.345	5.232		

1 Minimum extinction time.

2 Maximum extinction time.

3 Mean extinction time.

4 Standard Deviation.

5 Probability of observing extinction between weeks 18–28 PR, obtained from the outcomes of 1000 simulated experiments.

Results of mating competition experiments are summarized in Table 3. Mating competition experiments 1–5, which all used a 1∶1 OX3604C∶GDLS2 ratio, but varied in cage size and location, produced variable results as indicated by the significant heterogeneity statistic in the replicated G-test (Het. G = 38.04, p<0.01; see Table S4). However, even with this heterogeneity value, the pooled G-value (4.124) is significant (*p* = 0.042) and indicates a mean mating fitness cost of 10.8% for the OX3604C males (Table S4). Experiment 6 was designed to better reflect conditions in the field cage experiment with a 10∶1 OX3604C∶GDLS2 ratio and the same cages and initial density as the long-term experiments. Results of the replicates also were variable (Het. G = 20.72, *p* = 0.04) and the overall difference between the strains was significant (Pooled G = 43,83, *p*<0.01) with a mean mating fitness cost of 59.1% for OX3604C males (Table S4).

**Table 3:** Summary of mating competitiveness experiments.

experiment (# of replicate)		treatment (age of males)	type of cage^1^	Male ratio (OX3604C∶GDLS2)	Location^2^	# of GDLS2 batches	# of OX3604C batches	# of mixed batches	# of mixed batches (1∶1 ratio GDLS2∶OX3604C larvae)^3^	G test	*P* value
1		YOUNG OX3604C vs. YOUNG GDLS2	small cages	1∶1	field site	21	6	6	4	3.284	0.07
					insectary	6	23	3	2	11.136	<0.01*
2			small cages	1∶1	field site	8	18	1	1	3.793	0.05*
					insectary	19	6	2	2	3.793	0.05*
3			field cages	1∶1	field site	15	8	3	3	0.347	0.56
			small cages		insectary	13	8	4	3	0.042	0.84
4	(1)		field cages	1∶1	field site	41	32	4	3	0.329	0.57
			small cages			6	3	0	0	0.448	0.50
	(2)		field cages			46	18	6	6	6.398	0.01*
			small cages			7	2	3	3	0.083	0.77
5			field cages	1∶1	field site	47	23	7	4	4.933	0.03*
			small cages			9	8	1	1	2.055	0.15
			small cages		insectary	8	14	2	2	2.072	0.15
6	(1)	OLD OX3604C vs. YOUNG GDLS2	Cage 1	10∶1	field site	1	18	10	8	0.468	0.49
			Cage 4			7	9	11	8	6.472	0.01*
	(2)		Cage 1			7	17	6	4	4.927	0.03*
			Cage 4			11	12	2	1	20.034	<0.01*
	(1)	OLD OX3604C vs. OLD GDLS2	Cage 3			5	15	6	5	2.625	0.11
			Cage 8			2	20	5	5	<0.001	0.98
	(2)		Cage 3			4	19	6	2	0.356	0.55
			Cage 8			5	13	6	5	2.134	0.14
	(1)	YOUNG OX3604C vs. YOUNG GDLS2	Cage 2			7	17	6	5	4.6	0.03*
			Cage 7			6	15	1	0	5.18	0.02*
	(2)		Cage 2			7	16	4	4	5.276	0.02*
			Cage 7			3	11	3	3	0.556	0.46

1 In experiment 6 the cage number is specified.

2 At the field site location, small cages were placed in the field laboratory.

3 Number of mixed batches with the hypothesis of 1∶1 ratio of OX3604C∶GDLS2 larvae not rejected by Chi Square Test. For statistical analysis, this number was included in the number of OX3604C matings because we assumed they came from matings with heterozygous males and not from occasional double matings.

(*) = statistically significant results.

## Discussion

OX3604C males decreased, but did not eliminate target populations in the field-cage experiment. Experimental protocols were similar between the previous laboratory experiment [18] and our field cage experiment, including high 10∶1 release ratios that in both cases were expected, based on modeling, to favor population extinction. Over the course of the field cage study, the OX3604C∶target male ratio increased in all cages and reached the highest value of 1,000∶1 in cage 1 during week 17 post-release, but this did not result in extinction of the target population. These results also were consistent with the data on the frequency of the DsRed2 marker detected in larvae from treatment cages, which ranged from 20–54% during week 17 post-release when the trial was terminated. The high estimated OX3604C male fitness cost calculated from the field cage trial (97%), the lower than predicted population reduction, and the long estimated extinction times (average 23–65 weeks) lead to the expectation that male OX3604C may be less effective for population reduction under open field conditions than predicted from results of the laboratory cage experiment.

Output from a simulation model predicted that the lack of a homozygous OX3604C strain did not contribute significantly to the absence of population extinction, because of the high OX3604C∶target male release ratio. Presence of wild-type individuals in the transgenic population required that we manually sex pupae in order to avoid introducing wild-type females into treatment cages. This resulted in a difference in management of the transgenic strain relative to the target strain and relative to the handling of the transgenic strain by Wise de Valdez *et al* [18]. The mortality observed among the transgenic males before introduction into treatment cages was low (∼5%), suggesting that this additional handling did not cause substantial harm. Furthermore, transgenic male survival in the cages was high. As can be seen in Figure 3, there were on average about 5 times as many males in treatment cages as control cages on day six after each release of OX3604C. This is equivalent to a daily survival rate of ∼0.91, which is similar to published values for *Ae. aegypti* survival in houses in the field [24]. The potential effect of differential handling was not addressed directly by the mating competitiveness experiments because the need to separate males from females added an additional handling step for GDLS2 males and females, such that the treatment of the two types was matched more closely in these experiments than in the earlier field cage experiment. Because differences were not apparent in the field cage trial, male survival was not evaluated in mating competitiveness experiments.

Mosquito size is sometimes but not always [25] associated with fitness. For *Ae. aegypti*, Ponlawat and Harrington [26] reported greater mating success by larger than smaller males. Measurements made in the first two weeks of the field cage experiment indicated that OX3604C males (median wing length = 2.24) were slightly larger than GDLS2 males (median wing length = 2.17, Mann-Whitney U test *p*<0.01), so this is unlikely to have contributed to the field cage outcomes.

Results from mating competitiveness experiments with laboratory-reared mosquitoes in field cages have generally been found to underestimate fitness costs found when the same types of mosquitoes are released in the field [27]–[29]. While the mating disadvantage of OX3604C males observed in experiment 6 (59.1%) appears to be one factor explaining the lack of extinction in our field cage trial, if there had only been a fitness cost of 59%, some extinctions would have been expected (Figures S4, S5). Therefore, although short-term mating competitiveness experiments are useful in assessing one major component of fitness, they are not designed to measure as many aspects of fitness as are measured in long-term studies.

Adaptation of genetically-engineered mosquitoes and target populations to laboratory and field cages always needs to be taken into account when moving from the laboratory to the field. In the field cage trial, OX3604C was derived from introgression of the OX3604C construct into a GDLS1 genetic background, reared in laboratory conditions (i.e., stable temperature, relative humidity, and photoperiod) and mated in small, crowded laboratory cages for more than 20 generations, all of which potentially selected for capacity to mate in a small spaces, and other adaptations for increased fitness in a laboratory environment. Conversely, GDLS2 originated from mosquitoes collected from the same locations 2 years after those used to create GDLS1, and GDLS2 target populations were maintained in large outdoor field cages for 16 generations before the start of the experiment. During the prerelease period, GDLS2 populations experienced natural variation in daily temperature and relative humidity and mated successfully for ∼4 months in their large outdoor enclosures. Adaptation to field cages may have been an advantage for GDLS2 males when competing with OX3604C males for GDLS2 females.

Our results are consistent with those from previous mosquito studies [27], [28], [30] indicating that colony maintenance and mass rearing should be planned prior to field-cage or open-field trials. Rearing large numbers of transgenic mosquitoes in large outdoor, semi-field enclosures for several generations may help avoid undesirable laboratory adaptation and reduce fitness differences between transgenic mosquitoes and conspecifics in their natural, target populations. Short-term mating competition experiments in large field cages could be an efficient way to gather preliminary information on genetically-engineered mosquito fitness relative to local wild-type mosquitoes, but they only measure one important fitness component while field cage trials include additional components.

We emphasize the potential impact of differential strain adaptation to the field or laboratory, but it is also possible that the fitness difference was due to the transgenesis process. Although insertion of the transgene did not affect the ability of the OX3604C to cause extinction in the laboratory system, it is feasible that some negative pleiotropic effect of the gene insertion was manifested only under field cage conditions. Precautions were taken to avoid some negative effects that are often associated with transgenesis. Most importantly, the originally engineered strain was backcrossed for five generations to a strain for the local area where the experiment was conducted. This was expected to replace over 96% of the genes from the engineered strain with local strain genes, except for genes linked to the transgene. If the transgene had been inserted within a transcribed gene, it could have disrupted gene function that affected fitness in outdoor field cages, but not in the laboratory. Attempts to fine-scale map the location of the transgene indicated that the insertion was in a genomic area with repetitive DNA, indicating the transgene was not inserted within a transcribed gene.

Although an argument can be made for not pursuing an open field evaluation of OX3604C males based on our field cage results, the best way to resolve the discrepancy between laboratory and field cage results would be to assess them under uncontained, open field conditions. Because this has not been done, data do not exist to determine whether laboratory or field cage experiments are most informative about how this strain will perform under natural conditions. A different genetic background, different chromosomal location of the transgene or different rearing procedures could separately or in combination affect the competitiveness of transgenic mosquitoes. Evaluation of other *Ae. aegypti* strains carrying the female-flightless transgene would help determine if results observed in this trial apply to this genetic modification in general or are specific to the OX3604C strain we studied.

Our results support inclusion of large outdoor field cage experiments in the systematic, phased evaluation of GE *Ae. aegypti*, including those with transgenes like OX3604C that are self-limiting. Details of field cage construction and the level of containment needed will depend on the nature of genetic modification in the strain being evaluated as well as general requirements of the relevant regulatory authorities. If genetic modifications include the potential of elevated pathogen transmission or non-Mendelian inheritance (*i.e.*, genetic drive systems), strain evaluation will require higher security caging than those used in our experiments [20]. We stress that short-term mating competition experiments in large field cages could be used to obtain predictive information on mating competitiveness and fitness costs, but it is not clear that by themselves these would be sufficient substitutes for longer-term field cage tests. Results of appropriately planned, executed and analyzed open-field releases of the OX3604C would be useful in addressing this issue.

All of the work described here was conducted within ethical, social and cultural guidelines for community engagement activities [16]. We found that this approach helped us to develop respect and trust, basic ingredients for strong working relationships with local residents living near the field site, and for appropriate dialogue with state and national health and environmental authorities, scientists, and local and international press. Although the containment measures and communication activities taken in this work were greater than expected for research with natural strains of mosquitoes, we feel that this precautionary approach could have long-term benefits by decreasing suspicion that transgenic mosquito technology is being applied carelessly [31].

## Supporting Information

Figure S1 **Picture of the field cages.**
(PDF)

Figure S2 **Diagram of the field cage set up.** The OX3604C strain was reared in trays in the field laboratory (lower right). When adult males emerged they were moved to their corresponding treatment cage. Adults were sampled in cages using BG Sentinel Traps, transferred to the field laboratory, anesthetized in the CO_2_ sedation device, counted, sexed, and returned to the cage from which they came. For biosecurity, 10 BG Sentinel Traps and 10 oviposition traps were located around and below the platform, respectively.
(PNG)

Figure S3 **Each half cage contained two cabinets.** The top two shelves of each cabinet held two larval rearing containers covered by screened domes that prevented females from laying eggs (denoted by 1) and two oviposition containers (denoted by 2). A 15 L black plastic bucket partially covered with black plastic providing a sheltered and humid refugee (denoted by 3) and two plates with raisins (denoted by 4) provided a sugar source for adults.
(PNG)

Figure S4 **Simulated cage dynamics for varying combinations of percent homozygosity and fitness costs.** Heterozygotes have ½ of the fitness cost as homozygotes, and the fitness cost is assumed to occur at mating time. (Top) Simulated dynamics of eggs throughout the 17 week release period. (Bottom) Genotype frequency of OX3604C throughout the 17 week release period. Fitness costs are (a) 90%, (b) 80%, (c) 70%, and (d) 60%. Percentages of homozygosity are 100% (circles), 90% (squares), and 80% (triangles).
(PNG)

Figure S5 **Histograms of post-release extinction times, given in weeks, predicted by the model for different combinations of fitness costs and percent homozygosity.** Each row represents a different fitness cost while each column represents a different percentage of homozygosity. Heterozygotes have ½ of the fitness cost as homozygotes, and the fitness cost is assumed to occur at mating time.
(PNG)

Figure S6 **Device developed and used for mosquito sedation at the field site.** (A) There were four mesh-screened chambers, each accessible through two sleeved openings in the side of the table. (B) Carbon dioxide from a 40 L tank was regulated by a manometer and (C) its flow was piped into four screened containers, each one located in one of the four chambers. (D) Sedated mosquitoes were transferred to the mesh lid, counted, sexed, and returned to the cup and then to the field cage from which they came.
(PNG)

Figure S7 **Release ratios of OX3604C∶target males over time (log transformed data).** Ratios were estimated based on the number of OX3604C males added weekly to treatment cages and the weekly larval return rate.
(PNG)

Table S1 **Summary of differences between laboratory (Wise de Valdez** ***et al*** **.** [**18**] **) and field cage experiments near Tapachula, Mexico.**
(PNG)

Table S2 **Mean temperatures (±SD), maximum and minimum temperature, maximum and minimum daily temperature range, mean RH (±SD), and maximum and minimum RH recorded inside field cages and in a field outside of the cages.**
(PNG)

Table S3 **Number of adults collected with backpack aspirators in field cages when the experiment was terminated on week 17 PR and the ratio of females collected in control vs. treatment cages for each pair of cages.**
(PNG)

Table S4 **Total fitness cost (1-geometric mean of observed/expected OX3604C), calculated for mating competition experiments 1–5 and 6.**
(PNG)
