# Supplementary figures and images for: Field Cage Studies and Progressive Evaluation of Genetically-Engineered Mosquitoes
Source: PLoS Negl Trop Dis. 2013 Jan 17;7(1):e2001. doi: 10.1371/journal.pntd.0002001 (PMC3547837; doi:10.1371/journal.pntd.0002001)

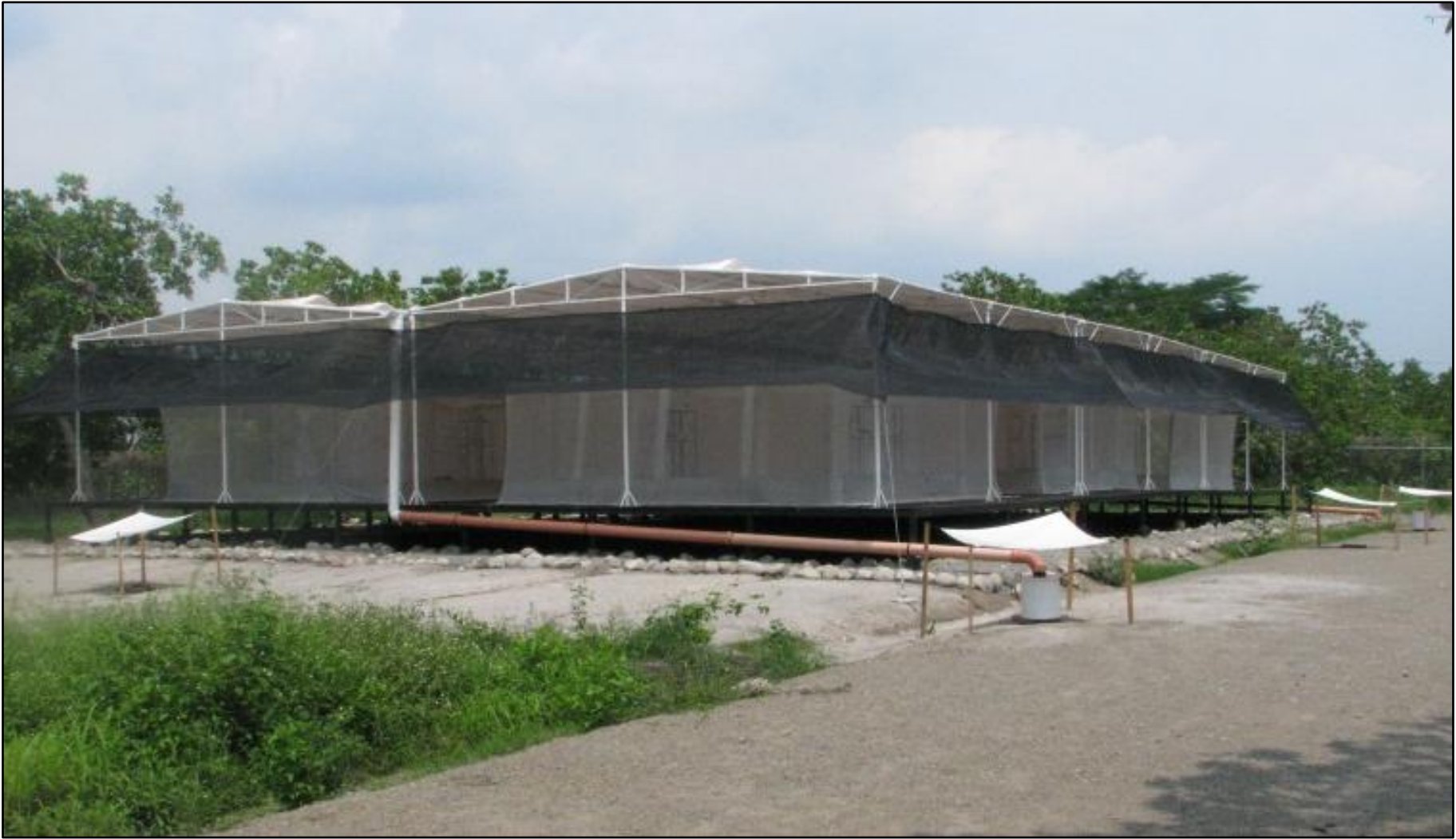

Supplement: Figure S1 — Picture of the field cages. (PDF) [file pntd.0002001.s001.pdf]

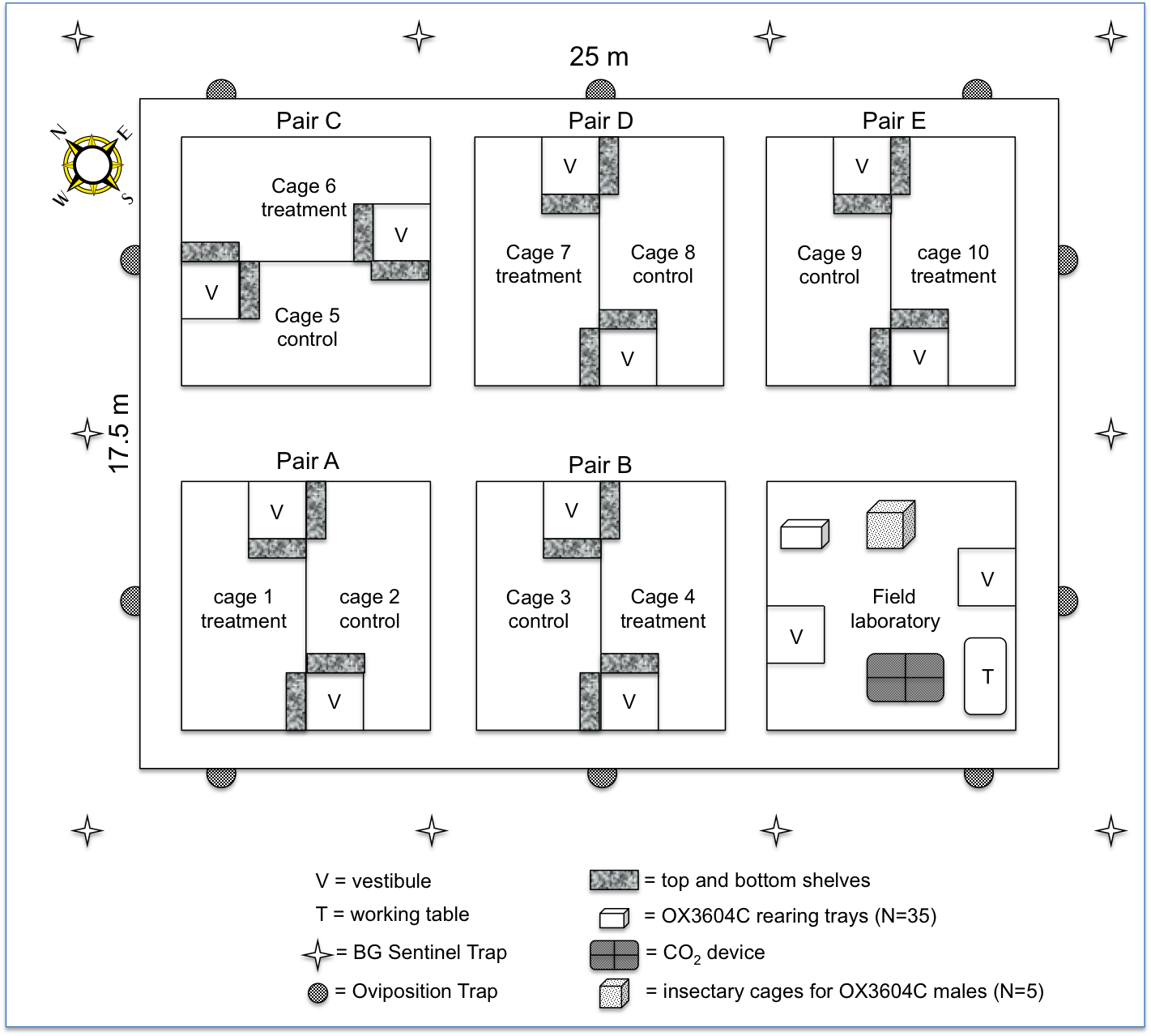

Supplement: Figure S2 — Diagram of the field cage set up. The OX3604C strain was reared in trays in the field laboratory (lower right). When adult males emerged they were moved to their corresponding treatment cage. Adults were sampled in cages using BG Sentinel Traps, transferred to the field laboratory, anesthetized in the CO2 sedation device, counted, sexed, and returned to the cage from which they came. For biosecurity, 10 BG Sentinel Traps and 10 oviposition traps were located around and below the platform, respectively. (PNG) [file pntd.0002001.s002.png]

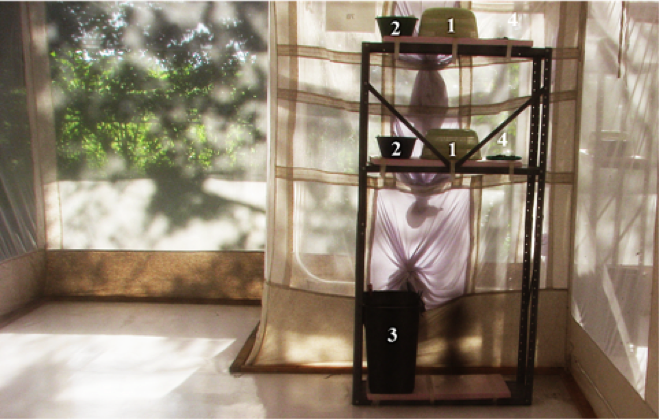

Supplement: Figure S3 — Each half cage contained two cabinets. The top two shelves of each cabinet held two larval rearing containers covered by screened domes that prevented females from laying eggs (denoted by 1) and two oviposition containers (denoted by 2). A 15 L black plastic bucket partially covered with black plastic providing a sheltered and humid refugee (denoted by 3) and two plates with raisins (denoted by 4) provided a sugar source for adults. (PNG) [file pntd.0002001.s003.png]

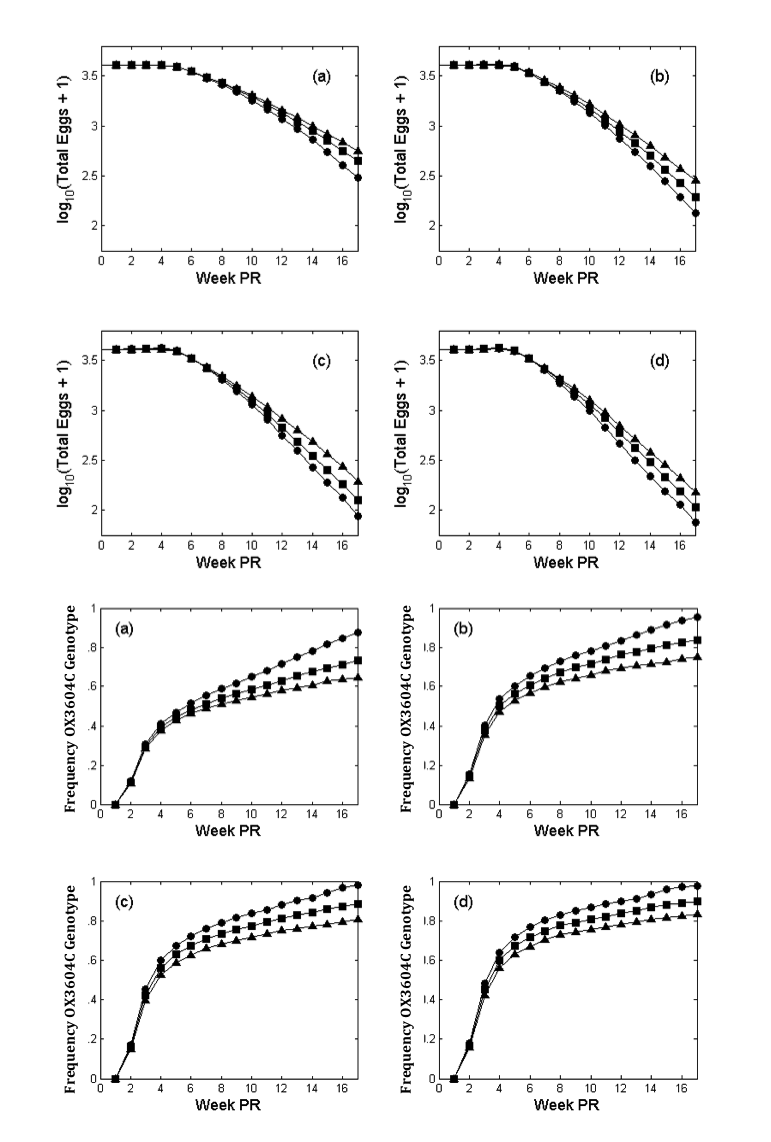

Supplement: Figure S4 — Simulated cage dynamics for varying combinations of percent homozygosity and fitness costs. Heterozygotes have ½ of the fitness cost as homozygotes, and the fitness cost is assumed to occur at mating time. (Top) Simulated dynamics of eggs throughout the 17 week release period. (Bottom) Genotype frequency of OX3604C throughout the 17 week release period. Fitness costs are (a) 90%, (b) 80%, (c) 70%, and (d) 60%. Percentages of homozygosity are 100% (circles), 90% (squares), and 80% (triangles). (PNG) [file pntd.0002001.s004.png]

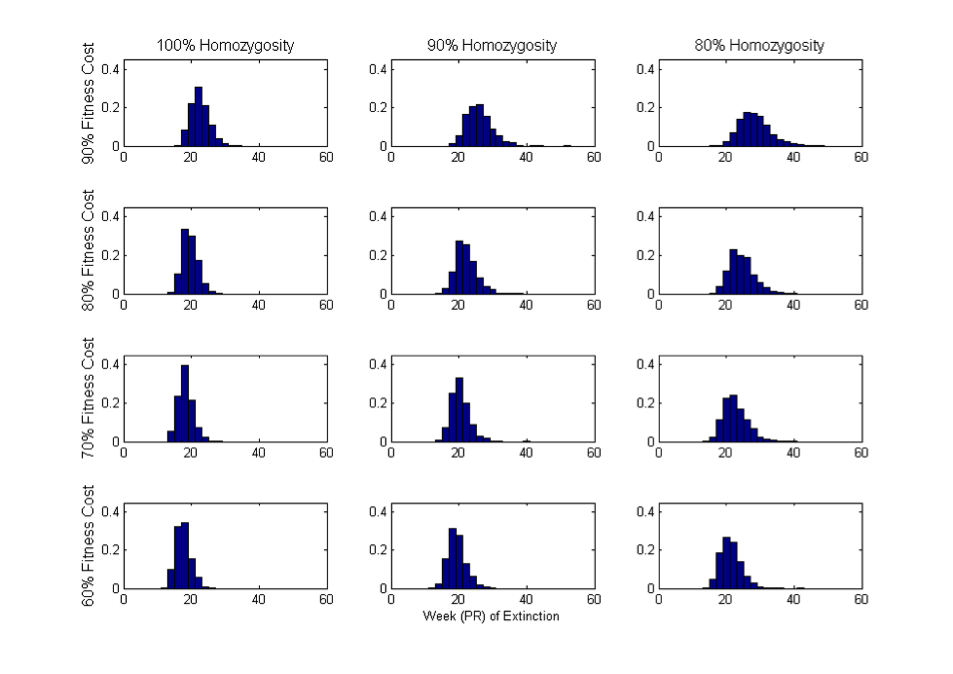

Supplement: Figure S5 — Histograms of post-release extinction times, given in weeks, predicted by the model for different combinations of fitness costs and percent homozygosity. Each row represents a different fitness cost while each column represents a different percentage of homozygosity. Heterozygotes have ½ of the fitness cost as homozygotes, and the fitness cost is assumed to occur at mating time. (PNG) [file pntd.0002001.s005.png]

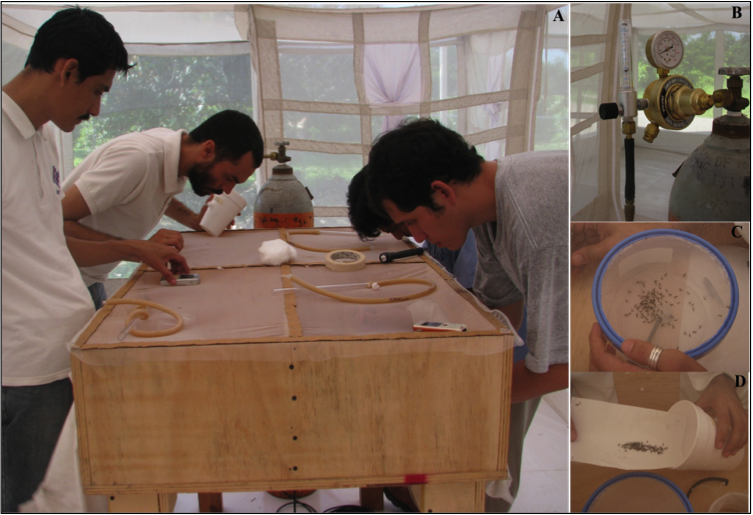

Supplement: Figure S6 — Device developed and used for mosquito sedation at the field site. (A) There were four mesh-screened chambers, each accessible through two sleeved openings in the side of the table. (B) Carbon dioxide from a 40 L tank was regulated by a manometer and (C) its flow was piped into four screened containers, each one located in one of the four chambers. (D) Sedated mosquitoes were transferred to the mesh lid, counted, sexed, and returned to the cup and then to the field cage from which they came. (PNG) [file pntd.0002001.s006.png]

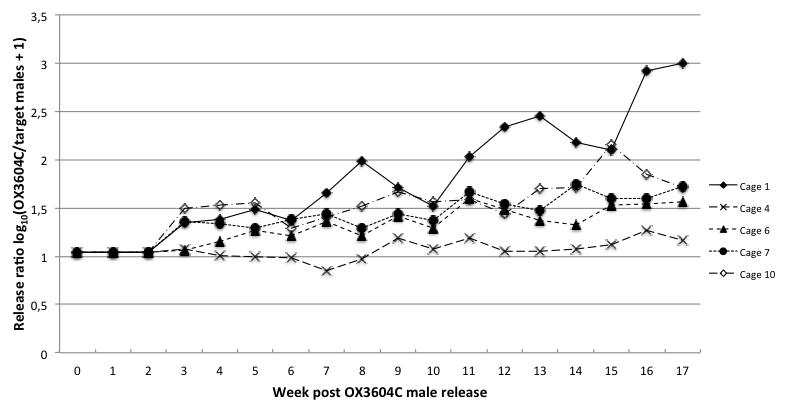

Supplement: Figure S7 — Release ratios of OX3604C∶target males over time (log transformed data). Ratios were estimated based on the number of OX3604C males added weekly to treatment cages and the weekly larval return rate. (PNG) [file pntd.0002001.s007.png]

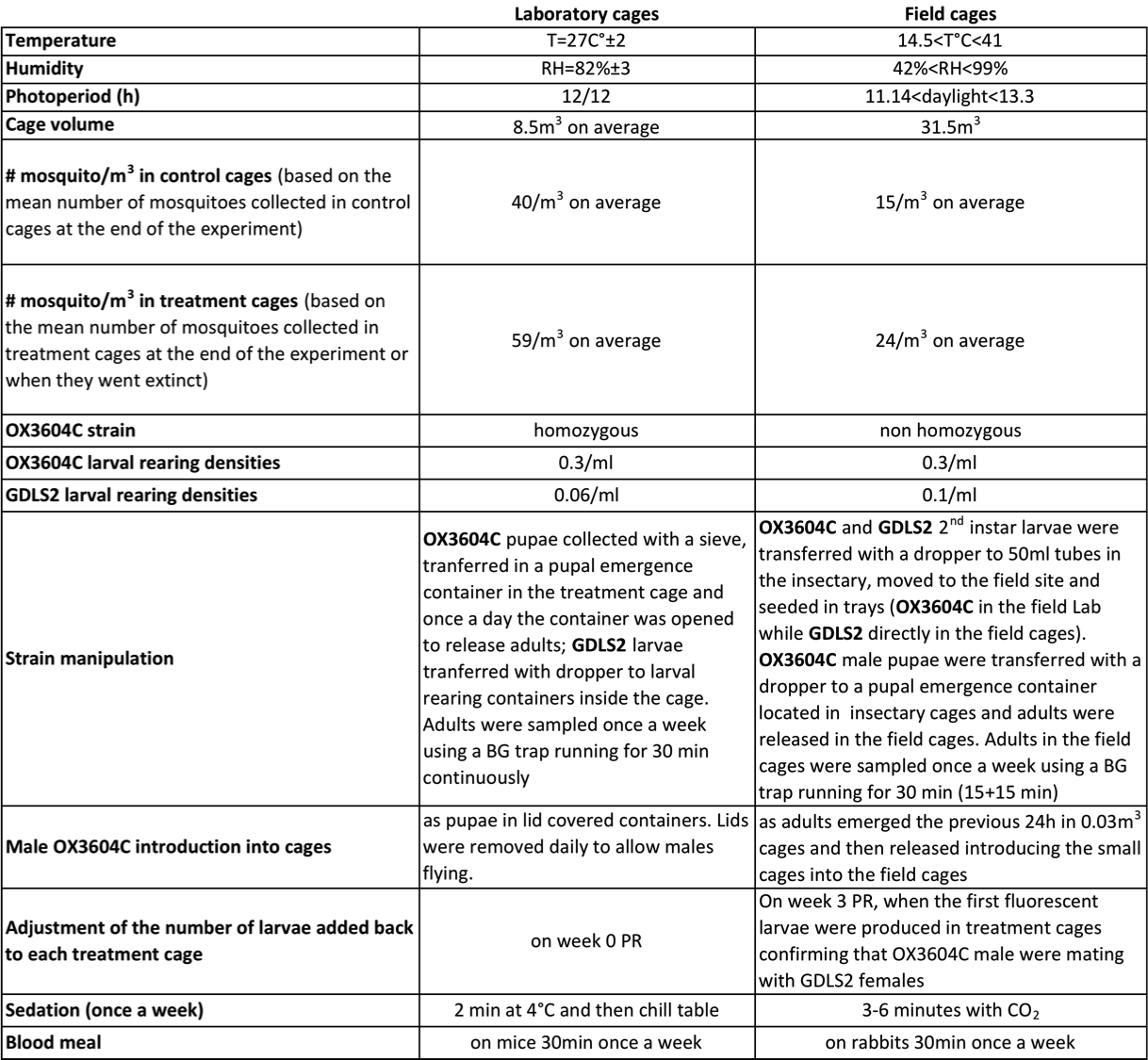

Supplement: Table S1 — Summary of differences between laboratory (Wise de Valdez et al . [18] ) and field cage experiments near Tapachula, Mexico. (PNG) [file pntd.0002001.s008.png]

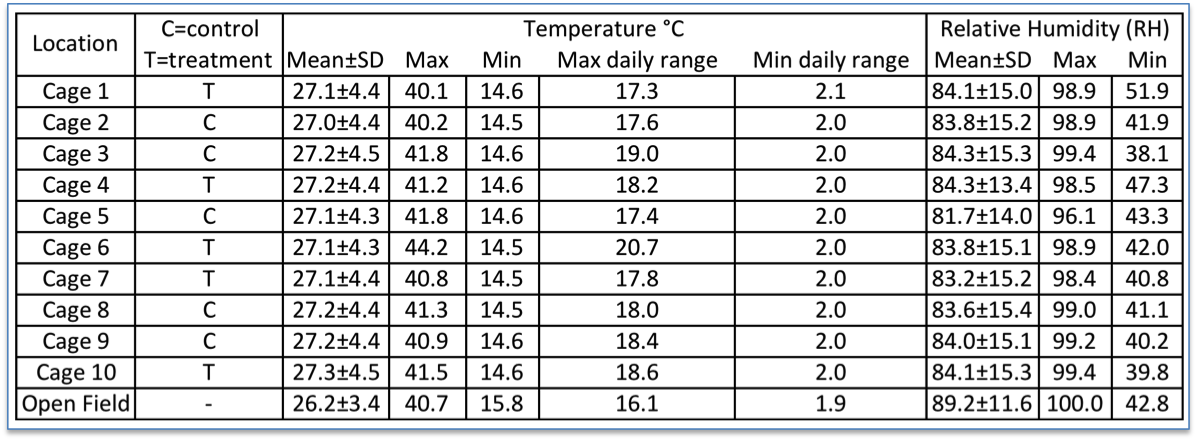

Supplement: Table S2 — Mean temperatures (±SD), maximum and minimum temperature, maximum and minimum daily temperature range, mean RH (±SD), and maximum and minimum RH recorded inside field cages and in a field outside of the cages. (PNG) [file pntd.0002001.s009.png]

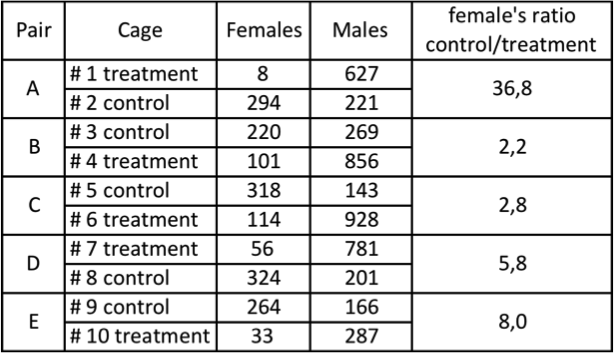

Supplement: Table S3 — Number of adults collected with backpack aspirators in field cages when the experiment was terminated on week 17 PR and the ratio of females collected in control vs. treatment cages for each pair of cages. (PNG) [file pntd.0002001.s010.png]

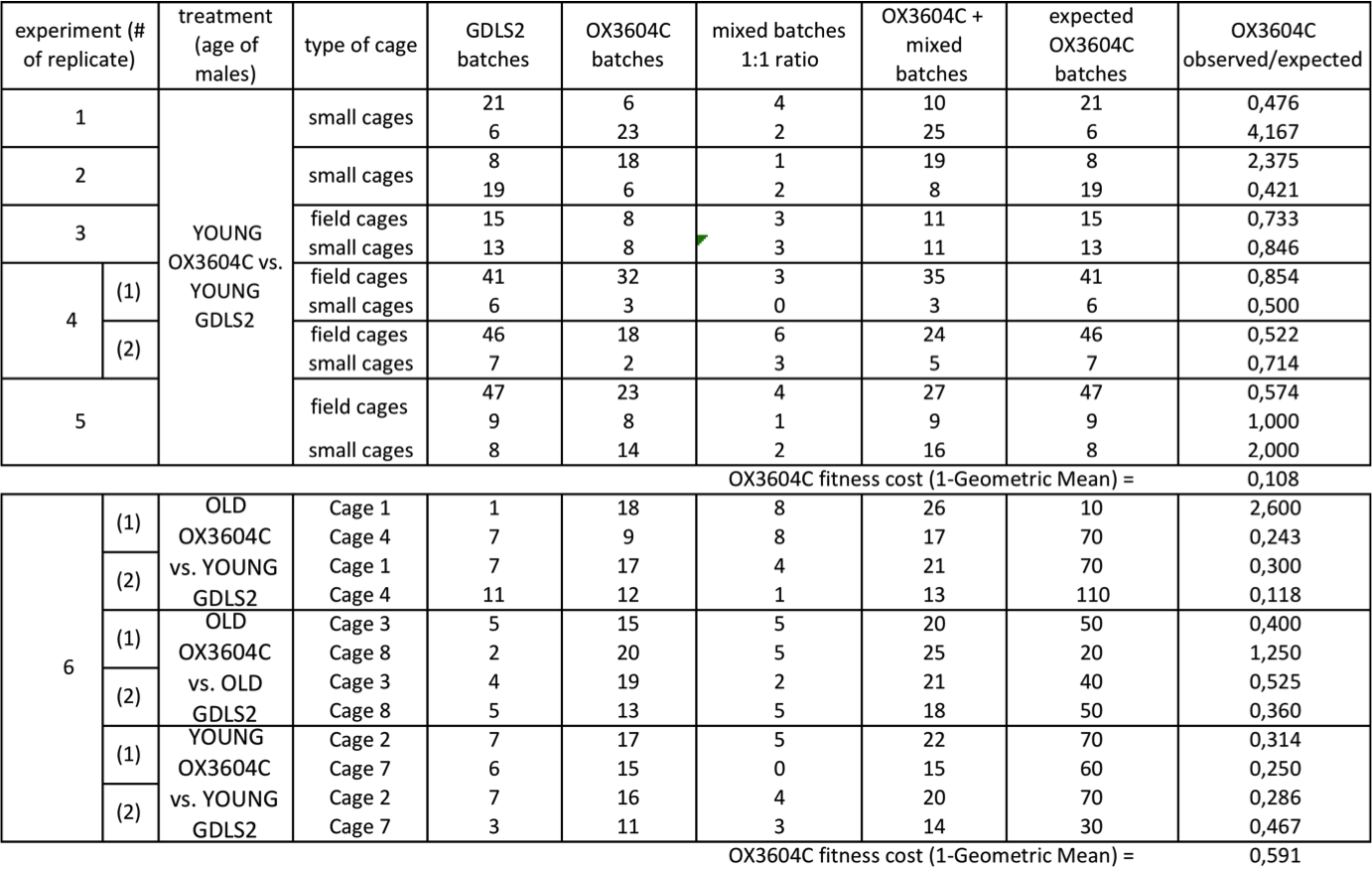

Supplement: Table S4 — Total fitness cost (1-geometric mean of observed/expected OX3604C), calculated for mating competition experiments 1–5 and 6. (PNG) [file pntd.0002001.s011.png]
